# Iron reduction by the deep-sea bacterium *Shewanella profunda* LT13a under subsurface pressure and temperature conditions

**DOI:** 10.3389/fmicb.2014.00796

**Published:** 2015-01-21

**Authors:** Aude Picard, Denis Testemale, Laura Wagenknecht, Rachael Hazael, Isabelle Daniel

**Affiliations:** ^1^Department of Biogeochemistry, Max Planck Institute for Marine MicrobiologyBremen, Germany; ^2^MARUM Center for Marine Environmental SciencesBremen, Germany; ^3^Center for Applied Geoscience, Eberhard Karls University TübingenTübingen, Germany; ^4^Institut Néel, Université Grenoble AlpesGrenoble, France; ^5^Institut Néel, Centre National de la Recherche ScientifiqueGrenoble, France; ^6^Christopher Ingold Laboratory, Department of Chemistry, University College LondonLondon, UK; ^7^CNRS, Laboratoire de Géologie de Lyon, Ecole Normale Supérieure de Lyon, Université Claude Bernard Lyon 1–Université de LyonUMR5276, Lyon, France

**Keywords:** Fe reduction, pressure, *Shewanella profunda*, temperature, XANES

## Abstract

Microorganisms influence biogeochemical cycles from the surface down to the depths of the continental rocks and oceanic basaltic crust. Due to the poor recovery of microbial isolates from the deep subsurface, the influence of physical environmental parameters, such as pressure and temperature, on the physiology and metabolic potential of subsurface inhabitants is not well constrained. We evaluated Fe(III) reduction rates (FeRRs) and viability, measured as colony-forming ability, of the deep-sea piezophilic bacterium *Shewanella profunda* LT13a over a range of pressures (0–125 MPa) and temperatures (4–37∘C) that included the *in situ* habitat of the bacterium isolated from deep-sea sediments at 4500 m depth below sea level. *S. profunda* LT13a was active at all temperatures investigated and at pressures up to 120 MPa at 30∘C, suggesting that it is well adapted to deep-sea and deep sedimentary environments. Average initial cellular FeRRs only slightly decreased with increasing pressure until activity stopped, suggesting that the respiratory chain was not immediately affected upon the application of pressure. We hypothesize that, as pressure increases, the increased energy demand for cell maintenance is not fulfilled, thus leading to a decrease in viability. This study opens up perspectives about energy requirements of cells in the deep subsurface.

## INTRODUCTION

Less than 1% of microorganisms of the environment have been isolated in pure cultures (Amann et al., 1995). This trend is even more pronounced for microorganisms living in the deep subsurface (D’Hondt et al., 2004; Parkes et al., 2009; Colwell and D’Hondt, 2013). For example, only a few tens of microorganisms have been isolated from deep subseafloor sediments (Mikucki et al., 2003; Biddle et al., 2006; Batzke et al., 2007). Therefore very little is known about the physiology of subsurface microorganisms. Pressure is a unique parameter of deep subsurface environments and increases at a rate of 10, 15, and 28 MPa km^-1^ in the water column, sediments and continental, and oceanic rocks, respectively. Piezophilic (i.e., pressure-adapted) microorganisms have their highest growth rate at pressures >0.1 MPa (Yayanos, 1995). Piezophilic bacteria and archaea have been isolated from seawater, subseafloor sediments, deep-sea animals, and wood fall (see review by Fang et al., 2010, for the most recent list of piezophilic microorganisms). The comparison between piezophilic and piezo-sensitive microorganisms has revealed physiological and molecular adaptations to life under pressure (Simonato et al., 2006; Lauro and Bartlett, 2007), such as a unique membrane composition of piezophilic bacteria (DeLong and Yayanos, 1985), an increase in certain amino acids in proteins of piezophilic archaea (Di Giulio, 2005) or pressure-regulated gene expression in some deep-sea bacteria (Bartlett et al., 1995; Nakasone et al., 1998).

Pressure and temperature have significant impact on microbial activity. Only a few anaerobic processes affecting biogeochemical cycles of the deep subsurface have been studied under pressure (Picard and Daniel, 2013, and references therein). Among those, sulfate reduction, which is the most important process in sedimentary subseafloor environments, is generally enhanced at *in situ* pressure (Kallmeyer and Boetius, 2004; Bowles et al., 2011; Vossmeyer et al., 2012). Although Fe(III) reduction is important in deep environments (Lovley and Chapelle, 1995), the effects of pressure and temperature on Fe(III) reduction have hardly been investigated. We previously showed that the bacterium *Shewanella oneidensis* MR-1 can proceed with Fe(III) reduction up to pressures of ∼110 MPa (Picard et al., 2012). While MR-1 is a piezo-sensitive strain, Fe(III) reduction rates (FeRRs) were increased in the range of 30–50 MPa (Picard et al., 2012). In *S. piezotolerans* WP3, FeRR, and magnetite production decreased with increasing pressure, concomitantly with an increase in crystallinity and grain size of magnetite (Wu et al., 2013).

In this study we investigated the effects of temperature and pressure on the FeRR and viability of *S. profunda* LT13a. The *Shewanella* group is appropriate to study the effects of pressure and temperature on metabolic processes as it includes species adapted to a variety of pressure and temperature regimes (Kato and Nogi, 2001). Moreover *Shewanella* is ubiquitous in the environment, making it a relevant model for environmental studies (Fredrickson et al., 2008). Finally *Shewanella* are metabolically diverse and several species of *Shewanella* have the ability to reduce Fe(III), allowing comparison of rates between species under similar conditions (Venkateswaran et al., 1999; Kato and Nogi, 2001). We show here that *S. profunda* LT13a is metabolically active over a large range of pressures (0–110 MPa) and temperatures (4–37°C). At high pressures (HPs), the respiratory chain does not seem immediately affected by pressure. We hypothesize that the increase in energy demand with pressure leads to a loss in viability and thus to an arrest of activity.

## MATERIAL AND METHODS

### BACTERIAL STRAIN AND CULTURE CONDITIONS

*Shewanella profunda* LT13a (named hereafter *Sp* LT13a) was purchased from the DSMZ collection (strain DSM 15900). *Sp* LT13a is a piezophilic and mesophilic bacterium isolated from deep-sea sediments, with optimal pressure and temperature for growth at 10 MPa and 30°C, respectively (Toffin et al., 2004). *Sp* LT13a has the ability to use Fe(III) as an electron acceptor (Toffin et al., 2004). *Sp* LT13a was grown aerobically in yeast extract-peptone (YP) medium at 30°C (shaking 160 rpm) to produce biomass and harvested after ∼15 h in early stationary phase, washed with saline solution and kept on ice until used. For iron reduction experiments, the minimal medium M1 was used (Kostka and Nealson, 1998) and supplemented with 2 gl^-1^ tryptone and 0.2 gl^-1^ yeast extract to provide electron donors and carbon sources. Fe(III)-citrate was prepared as previously described (Kostka and Nealson, 1998) and added to the medium at a final concentration of 3 and 5 mM, for pressure and temperature experiments, respectively. *Sp* LT13a was inoculated in M1 medium at initial CFU concentrations of ∼10^8^ cells ml^-1^ for temperature experiments and of ∼10^8^ and ∼10^9^ cells ml^-1^ for pressure experiments, then the inoculated medium was distributed to incubation vessels (see below). In our study, a high-density inoculum was used to obtain the most from the limited amount of beamtime available at the synchrotron (see subsection on *in situ* pressure experiments) and to study the effects of pressure on initial FeRR without significant variations of the cell density at the beginning of the experiments. The use of high-density inocula is also typical of iron reduction experiments (Roden and Zachara, 1996; Roden, 2006).

Fe(III) reduction at atmospheric pressure was investigated at 4, 10, 20, 30, and 37°C, in a series of mini hungate tubes closed with stoppers and plastic screw caps. All mini hungate tubes are thus individual experiments started from the same cell suspension. At each time point, one mini hungate tube was taken and its content was used for Fe(II) measurements using the ferrozine assay (Stookey, 1970) and for colony-forming unit (CFU) counts. For pressure incubations at 30°C, cultures were inoculated in serum bottles closed with rubber septa and sealed with aluminum caps then transferred to containers that fit in the two different pressure vessels used for these experiments (see next sub-sections). For each pressure experiment, the leftover culture was incubated at atmospheric pressure in the serum bottle as a control. Headspace in hungate tubes and serum bottles was flushed with N_2_ to ensure anoxic conditions. Abiotic controls were performed using medium without cells.

### *EX SITU* PRESSURE EXPERIMENTS (10–50 MPa)

Cultures were transferred from serum bottles to sterile plastic syringes that were sealed with a needle inserted in a rubber stopper after removing the headspace. All syringes represent individual experiments started from the same cell suspension. Syringes were incubated in HP cylinders similar to those described by Zobell and Oppenheimer (1950). Pressure was transmitted to the culture via the syringe piston in a HP cylinder filled with water as pressure-transmitting medium, pressurized using a multi-fluid hand pump (MP-1000, Enerpac^TM^). The maximal pressure achieved in the HP cylinders was 50 MPa. HP cylinders filled with water were stored and pre-incubated at 30°C to minimize pressure changes. Several syringes were incubated in each HP cylinder. At each sampling time, the HP cylinders were decompressed to recover one syringe then recompressed. Similarly to the ambient pressure experiments, the content of the syringes was used for Fe(II) measurements and CFU counts.

### *IN SITU* PRESSURE EXPERIMENTS (10–125 MPa)

To avoid decompression at each sampling time, we employed an experimental setup that enables *in situ* monitoring of Fe oxidation state and speciation in microbial cultures in an optimized pressure incubation system (autoclave) using X-ray absorption spectroscopy. The feasibility of this method was validated in a previous work (Picard et al., 2011). The experimental setup used in this study is the same as the one used for the study of Fe(III) reduction under pressure by *S. oneidensis* MR-1 (Picard et al., 2012). For each pressure experiment, a culture was prepared as described above and transferred from a serum bottle to the sample container, which is a glass-like carbon tube closed at both ends by free pistons. The glass-like carbon tube was then quickly transported to the beamline and placed inside the autoclave, which is aligned within the X-ray beam path and in front of detectors (Testemale et al., 2005). The autoclave that was thermalized at 30°C through the duration of all experiments was then pressurized to a given pressure between 10 and 125 MPa. The HP windows of the autoclave were made of 0.5 mm glass-like carbon for entrance and exit ports and of 4.5 mm Be for the fluorescence port, to maximize the signal at the low X-ray energy of the Fe absorption edge (7112 eV). The Fe oxidation state was monitored *in situ* in the autoclave without decompression by acquiring at regular time intervals X-ray absorption near-edge structure (XANES) spectra at the Fe K-edge. The culture was decompressed only at the end of the experiments and was recovered for CFU counts. With this experimental setup, only one experiment can be performed at a time. The use of high-density inoculum allowed us to optimize the number of experiments during the allocated beamtime and perform experiments at 10, 60, and 80 MPa during a first session and at 10, 40, 70, 110, 125 MPa during a second session. Only the 10-MPa experiment was duplicated. After filling the glass-like carbon tube, the culture left in the serum bottle was incubated at 30°C and atmospheric pressure and served as a control to ensure that the strain behaved similarly (i) from one experiment to another and (ii) from one experimental session at the synchrotron to another and (iii) and that the changes observed in Fe(II) production were caused by the sole pressure parameter. Abiotic controls consisted of medium free of cells incubated at 30°C and atmospheric pressure within which the Fe oxidation state was monitored with similar acquisition parameters. No photoreduction of Fe(III) occurred under the X-ray beam.

### FERROZINE ASSAY

Samples (100 μl) were acidified in 900 μl 0.5 M HCl and Fe^2+^ was quantified using the ferrozine assay (Stookey, 1970).

### X-RAY ABSORPTION NEAR-EDGE STRUCTURE SPECTROSCOPY AND DATA ANALYSIS

X-ray absorption near-edge structure spectroscopy was performed at the FAME beamline of the European Synchrotron Radiation Facility (Grenoble, France) during two experiments for a total of 10 days (6 days allocated in 2010 and 4 days allocated in 2012). The optical characteristics of the beamline are described elsewhere (Proux et al., 2005, 2006). XANES spectra were acquired at the Fe K-edge at regular time intervals in the autoclave. Details on the acquisition parameters are available in Picard et al. (2012). XANES spectra were processed and analyzed using the Horae Athena software (Ravel and Newville, 2005). Each XANES spectrum was normalized and fitted to a linear combination of Fe(II) and Fe(III) standard spectra as measured in solutions of Fe(II) sulfate and Fe(III) citrate, respectively, using a least-square minimization procedure. From the relative contribution of Fe(II) and Fe(III) at each time point and from the known initial concentration of Fe(III) in the medium, we could calculate Fe(II) concentrations as a function of time.

### COLONY-FORMING UNIT COUNTING

The CFU assay was used to estimate the number of viable cells at the end of *in situ* experiments, at each sampling point during *ex situ* experiments, as well as on controls of the *in situ* experiments. 100 μl of culture was serially diluted in saline solution (NaCl 0.9%) and 4 drops of 10 μl from each dilution were deposited on solid M1 medium containing Fe(III)-citrate. Colonies were counted after 2 days of incubation at 30°C. Culture samples from the autoclave were plated after 12 h to allow for recovery.

### KINETIC MODEL

Similarly to our previous study on the effects of pressure on FeRR by *S. oneidensis* MR-1 model (Picard et al., 2012), the data were always fitted by a first-order kinetic model:

$${Fe\left(II\right)}_{t}={Fe\left(II\right)}_{\max}\left(1-e^{-kt}\right)$$

where Fe(II)*_t_* is the concentration of Fe(II) in mM at time *t*, Fe(II)_max_ is the final concentration of Fe(II) produced [equivalent to the maximal amount of Fe(III) reduced] in mM, *k* is the first-order rate coefficient in h^-1^ and *t* is the time in h. Initial FeRRs in mM h^-1^, referred to as FeRR were calculated at *t* = 0. Average cellular FeRR were calculated for *Sp* LT13a, for *S. oneidensis* MR-1 based on our previous study (Picard et al., 2012) and unpublished results, and recalculated for *S. piezotolerans* WP3 (Wu et al., 2013) by dividing initial FeRR by initial CFU concentrations.

## RESULTS

### EFFECTS OF TEMPERATURE ON IRON REDUCTION

**FIGURE 1:**
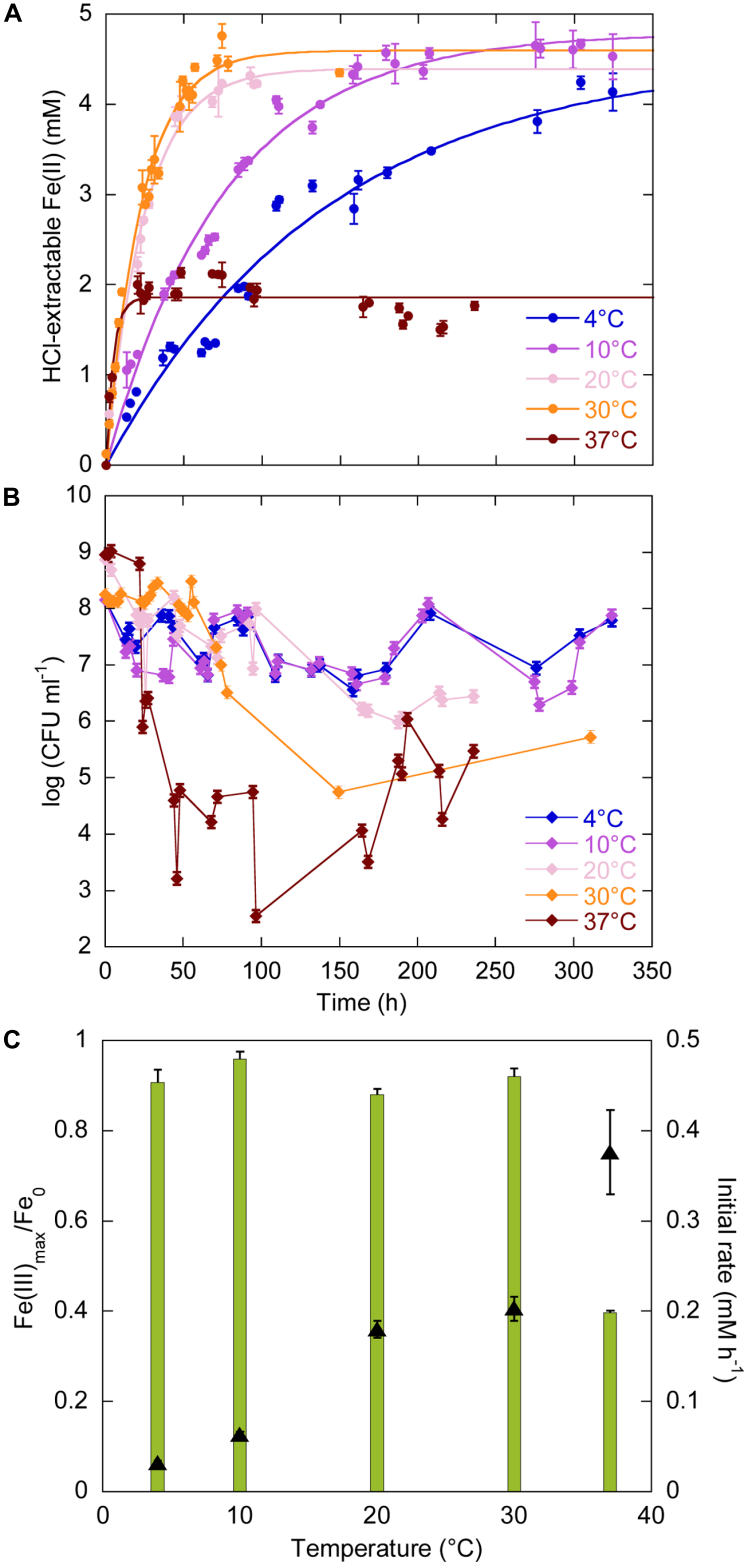
**Effects of temperature (4, 10, 20, 30, and 37°C) on Fe(III) reduction and colony-forming ability by *Shewanella profunda* LT13a. (A)** Fe(II) production as a function of time as measured with the ferrozine assay. Each data point represents the Fe(II) concentration measured in an individual culture and the error bar is the SD of two measurements. Solid lines represent first-order kinetic fits of the data. **(B)** CFU concentrations as a function of time (represented as log). Error bars represent standard deviation of the CFU counts. **(C)** Fe(III) reduction rates (FeRR, black triangles, right axis) and Fe(III) reduction extent (green bars, left axis) as a function of temperature. Error bars represent errors from the kinetic fits.

The temperature range for growth of *Sp* LT13a is 4–37°C (Toffin et al., 2004). We investigated the effects of temperature on iron reduction by strain LT13a at 4, 10, 20, 30, and 37°C at atmospheric pressure (**Figure 1**). At all temperatures Fe(III) reduction started immediately after the beginning of the experiments and Fe(II) production reached a plateau (**Figure 1A**). At 20, 30, and 37°C, CFU concentrations were stable or slightly oscillating until just before Fe(II) reached a plateau, after 100, 60, and 25 h, respectively, then dropped to 10^6^, 10^5^, and 10^3^ CFU ml^-1^, respectively (**Figure 1B**). After 180, 150, and 100 h, respectively, CFU concentrations increased again to reach 10^5^–10^6^ CFU ml^-1^ (**Figure 1B**). At 4 and 10°C, CFU concentrations oscillated as a function of time but never fell below 10^6^ CFU ml^-1^. At 4, 10, 20, and 30°C, almost all Fe(III) provided was reduced, i.e., 91, 96, 88, and 92%, respectively (**Figure 1C**, green bars, left axis). The amount of Fe(III) reduced at 37°C decreased to 40% of the total Fe(III) provided (**Figure 1C**, green bars, left axis). FeRR increased linearly with temperature between 4 and 37°C (**Figure 1C**, black triangles, right axis). Although the highest initial rate at 37°C was almost twice as high as the rate at 30°C, the cells stopped reducing Fe(III) after 25 h. A linear regression analysis of the FeRR plotted according to the Arrhenius equation allowed to calculate an activation energy of 50.3 ± 0.8 kJ mol^-1^ for Fe(III) reduction (data not shown).

### EFFECTS OF PRESSURE ON IRON REDUCTION

**FIGURE 2:**
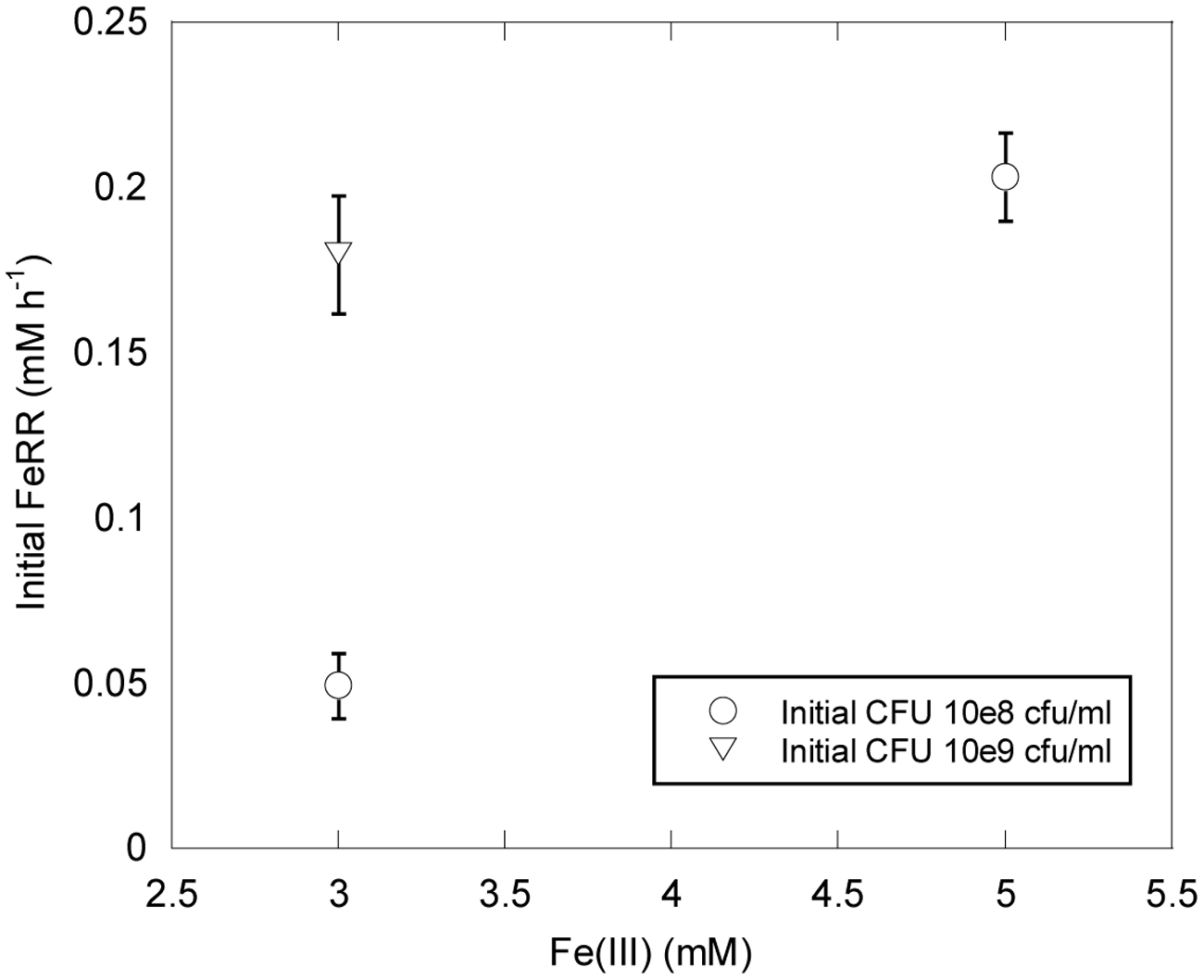
**Effects of Fe(III) concentration and initial CFU on FeRR at atmospheric pressure and 30°C.** Error bars represent errors from kinetic fits.

*Shewanella profunda* LT13a grows in the range 0–50 MPa (Toffin et al., 2004). In this study we used initial CFU concentrations of ∼10 and ∼10^9^ CFU ml^-1^ higher than those typically used for growth experiments. We could thus evaluate the effect of pressure on initial FeRR without variation of cell concentration during the first stage of incubations. At atmospheric pressure, initial FeRR increased with initial CFU concentration (**Figure 2**). At similar initial CFU concentrations, FeRR were lower when 3 mM Fe(III) was provided as initial electron acceptor concentration than when 5 mM Fe(III) was provided (**Figure 2**).

**FIGURE 3:**
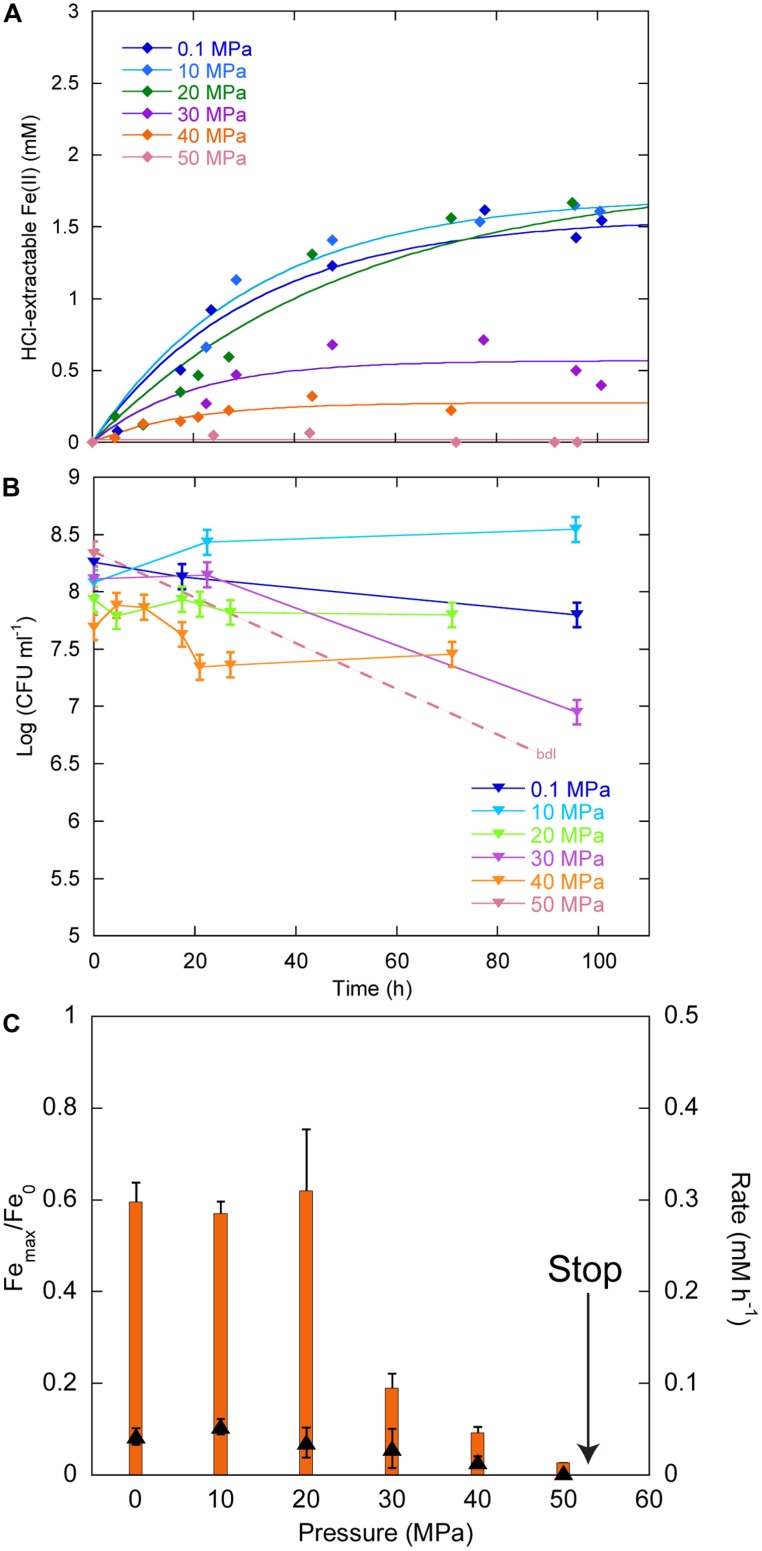
**Effects of pressure on iron reduction by LT13a with an initial cell concentration of ∼10^8^ CFU ml^-1^. (A)** Fe(II) production as a function of time as measured with the ferrozine assay. Each data point represents the Fe(II) concentration measured in an individual culture. Solid lines are first-order kinetic fits of the data. **(B)** CFU concentrations as a function of time (represented as log). Error bars represent SD of the CFU counts. bdl indicates “below detection limit.” **(C)** Fe(III) reduction rates (FeRR, black triangles, right axis) and Fe(III) reduction extent (orange bars, left axis) as a function of pressure. The “stop” arrows points to the pressure limit of 53 MPa extrapolated from data. Error bars represent errors from the kinetic fits.

**Figure 3A** displays the Fe(II) production as a function of time in cultures inoculated with ∼10^8^ CFU ml^-1^. Between 0.1 and 40 MPa, no delay was observed in the production of Fe(II), while initial Fe(II) production was very limited at 50 MPa. Over the first 20 h, CFU were stable at 0.1, 20, and 30 MPa, increased at 10 MPa and decreased at 40 MPa (**Figure 3B**). At 50 MPa, no CFU could be counted after the start of the experiment. Cells reduced 60% of Fe(III) provided at 0.1, 10, and 20 MPa, 19, 9, and 3% at 30, 40, and 50 MPa, respectively (**Figure 3C**, orange bars, left axis). FeRR were the highest at 10 MPa and then decreased with increasing pressure (**Figure 3C**, black triangles, right axis). A linear fit to the iron reduction yield between 10 and 50 MPa (not shown) indicated that *Sp* LT13a cells stopped reducing Fe(III) at 53 ± 2 MPa.

**FIGURE 4:**
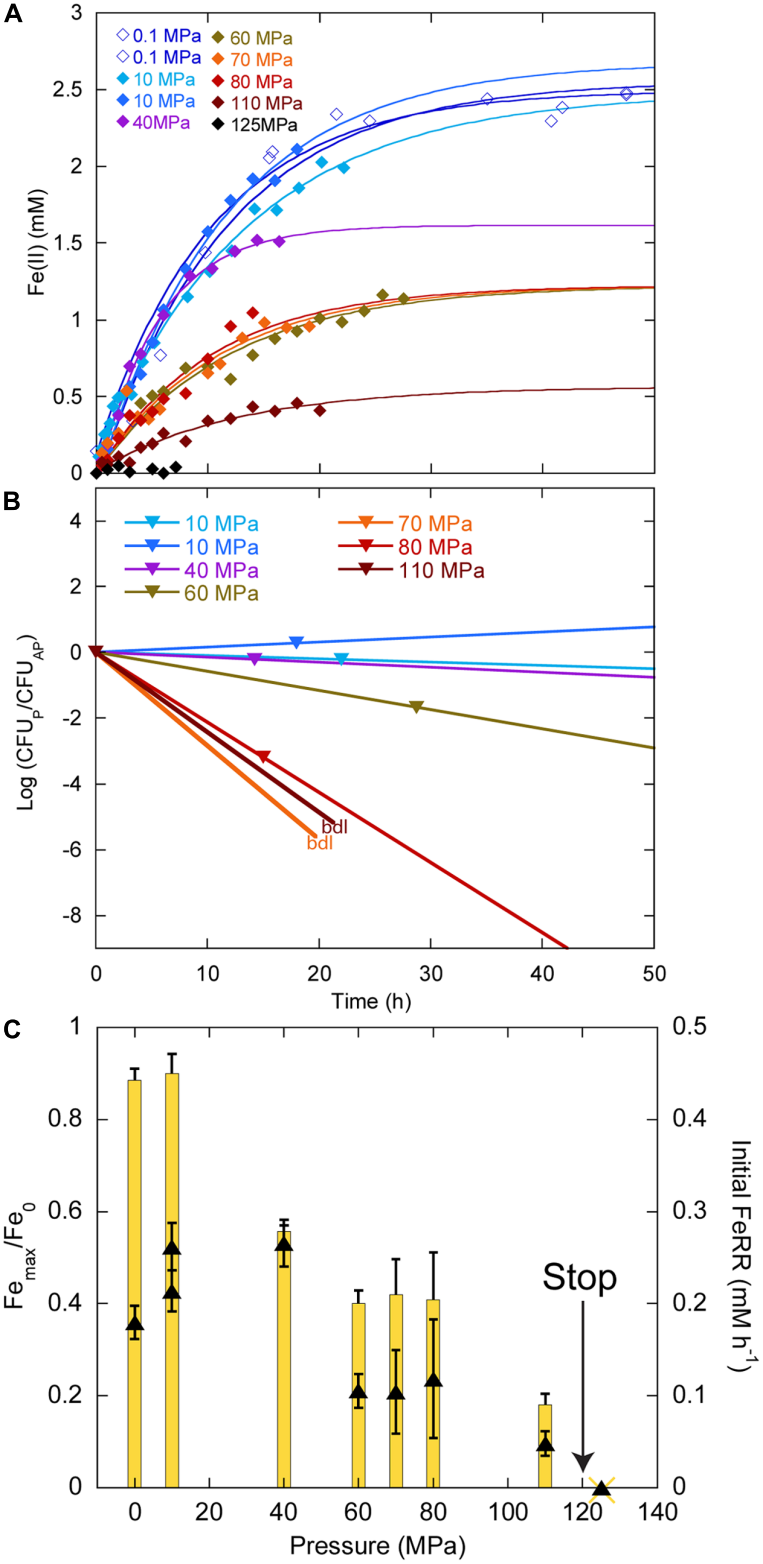
**Effects of pressure on iron reduction by LT13a with an initial cell concentration of ∼10^9^ CFU ml^-1^. (A)** Fe(II) production as a function of time calculated from linear combination fitting analysis of XANES spectra (filled diamonds). Error bars, representing the error on the linear combination fits, are smaller than symbols. Fe(II) measurements at 0.1 MPa were performed EX SITU with the ferrozine assay (open diamonds). Solid lines are first-order kinetic fits of the data. **(B)** CFU concentrations as a function of time (represented as log of CFU measured at the end of the experiments normalized by the CFU measured at the end of the control experiment at atmospheric pressure, see Materials and Methods). bdl indicates “below detection limit.” **(C)** Fe(III) reduction rates (FeRR, black triangles, right axis) and Fe(III) reduction extent (yellow bars, left axis) as a function of pressure. The “stop” arrows points to the pressure limit of 120 MPa extrapolated from data. Error bars represent errors from the kinetic fits.

In experiments starting with the highest CFU concentration 10^9^ CFU ml^-1^, we did not observe any delay in Fe(II) production between 0.1 and 110 MPa (**Figure 4A**). At 125 MPa, no Fe(III) was reduced anymore. Up to 40 MPa, CFU concentrations at the end of experiments were close to the initial ones, whilst CFU concentrations decreased between 60 and 80 MPa (**Figure 4B**). At the end of the experiments at 70 and 100 MPa, there were no CFU forming anymore. Unfortunately, the culture incubated at 125 MPa was lost during the decompression of the autoclave, as it occurs sometimes when helium that serves as pressure medium does not exsolve fast enough from either the medium or the seals. Cells reduced 88% of the Fe(III) provided at atmospheric pressure (**Figure 4C**, yellow bars, left axis). The highest amount of Fe(III) reduced was 90% at 10 MPa. This amount decreased progressively with increasing pressure. At 110 MPa, cells still reduced 18% of the Fe(III) provided. FeRR were highest between 10 and 40 MPa then decreased as pressure increased. A linear fit to the data between 40 and 100 MPa (not shown) estimated the arrest of activity at 120 ± 5 MPa, in good agreement with the experiment at 125 MPa that did not show any activity.

### AVERAGE CELLULAR FE(III) RESPIRATION RATES UNDER PRESSURE

**FIGURE 5:**
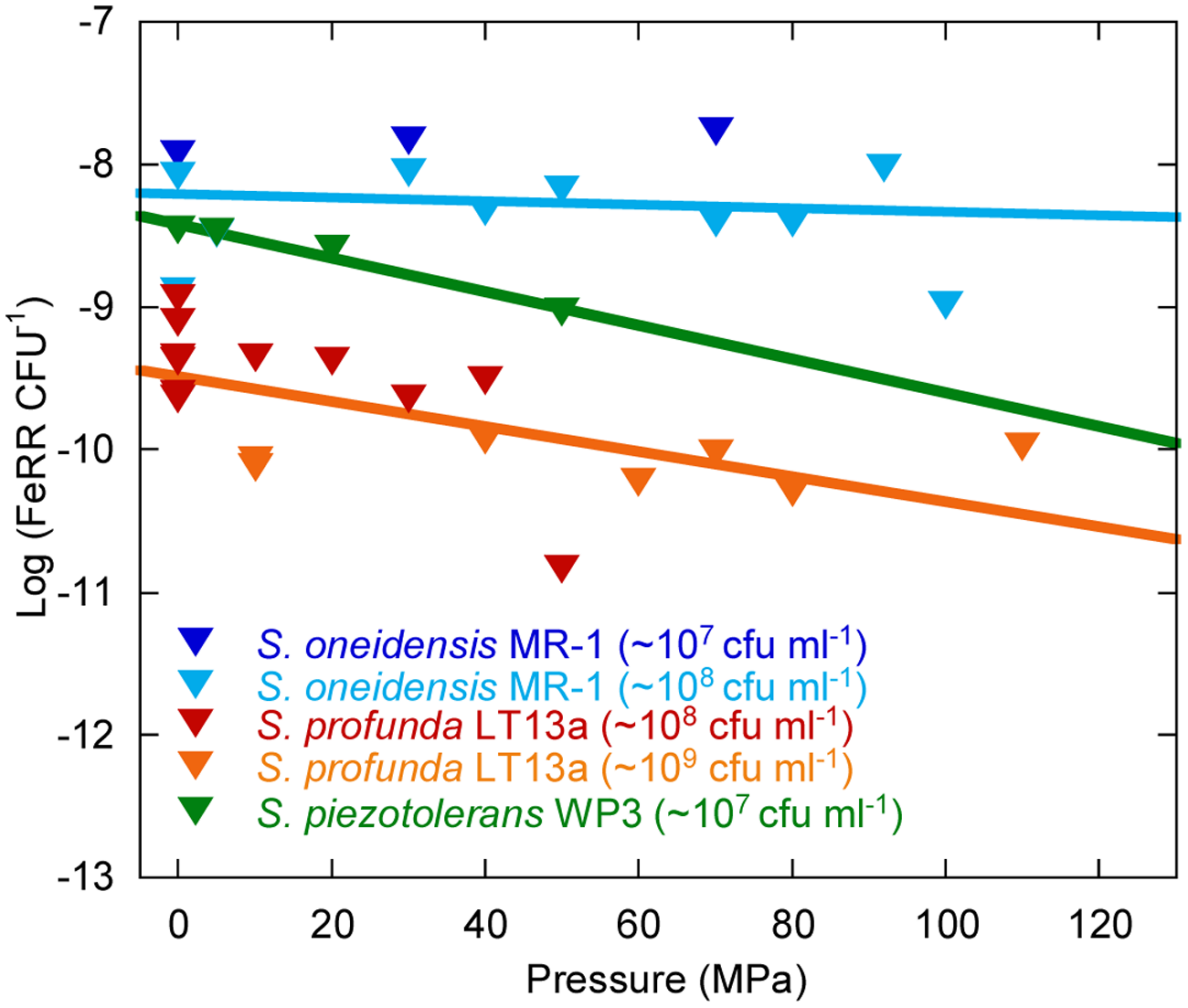
**Influence of pressure on the average cellular Fe(III) reduction rates (initial FeRR CFU^–1^).** Data for *S. oneidensis* MR-1 (dark and light blue triangles) are re-plotted from Picard et al. (2012) and Picard (unpublished work). Data for *S. piezotolerans* WP3 (dark green triangles) are recalculated from Wu et al. (2013). Data for *S. profunda* LT13a are from this study (red and orange triangles). Solid lines are linear fits to the data. The initial CFU concentration used in experiments is indicated in the legend.

We evaluated the effects of pressure on average initial FeRR per CFU in *Sp* LT13a (**Figure 5**). All values fell in the same range, independently of the initial CFU concentration. A linear regression analysis of the cellular FeRR showed only a slight decrease as a function of pressure. We compared these results with average cellular FeRR of *S. oneidensis* MR-1, based on our previous study (Picard et al., 2012) and of *S. piezotolerans* WP3 (Wu et al., 2013). Average cellular FeRR were higher in *S. oneidensis* MR-1 than in *S. profunda* LT-13. *S. piezotolerans* WP3 fell between both strains. Cellular FeRR decreased at the same rate for both deep-sea strains while these were stable over the *P* range of activity in MR-1.

## DISCUSSION

### CELL DENSITY AFFECTS P RANGES OF ACTIVITY AND SURVIVAL

At ambient pressure, FeRR increased with increasing initial CFU concentration (**Figure 2**), as seen in other studies with *S. alga* BrY (Roden and Zachara, 1996) and *Geobacter sulfurreducens* (Roden, 2006). The upper pressure limit for Fe(III) reduction and colony-forming ability appear to be dependent on the initial cell concentration (**Figures 3** and **4**). At both initial cell concentrations, the arrest of activity is correlated with a loss of viability. The higher pressure limit in experiments starting with 10^9^ CFU ml^-1^ could be explained by the fact that more cells are available to reduce Fe(III) before pressure affects the vitality of cells. This is in good agreement with a previous study investigating the inactivation of *Escherichia coli* at 100 MPa and 45°C with varying initial cell concentration. The inactivation rate of *E. coli* by pressure decreases with the increase of initial cell concentration from 10^4^ to 10^9^ cells ml^-1^ (Furukawa et al., 2002). One hypothesis to explain this observation is that cells might form aggregates at high cell concentration that are more resistant to pressure than cells alone (Furukawa et al., 2002). The presence of quorum sensing in *Shewanella* has been suggested by the presence of signaling molecules in several *Shewanella* species, including marine species and strain MR-1 (Bodor et al., 2008). In *Sp* LT13a, high CFU concentrations could induce changes in the metabolic state of cells, for example by channeling most energy produced via Fe(III) reduction to cell-maintenance-related processes, as opposed to growth. The amount of energy used for processes related to growth is higher than that of processes related to cell maintenance or survival (Hoehler and Jorgensen, 2013).

### PRESSURE RANGE FOR GROWTH IS NOT INFLUENCED BY CELL DENSITY

In our experiments, the initial CFU concentrations were similar to those that are reached in stationary phase after an exponential growth phase. Although the metabolic state of cells was certainly different than in an “usual” stationary phase, the observed changes in CFU were similar to those observed in *E. coli* in a long-term stationary phase (Finkel, 2006). CFU levels were indeed maintained at stable levels at atmospheric pressure, while oscillations occurred, indicating that cells were in a steady-state over the course of the experiments, with a death rate roughly equivalent to the growth rate (**Figures 3B** and **4B**). Under pressure, CFU were stable up to 40 MPa, at both initial cell concentrations, while they decreased quickly above 50 MPa, indicating that the death rate rapidly exceeded the growth rate (**Figures 3B** and **4B**). This is in good agreement with the original description of *Sp* LT13a that grew up to 50 MPa (Toffin et al., 2004). As a comparison, biosynthetic processes, e.g., cell division or DNA replication, are inhibited in this pressure range in *E. coli* (Bartlett, 2002).

### MECHANISMS OF MICROBIAL ACTIVITY INHIBITION BY PRESSURE

Upon application of pressure, no delay in Fe(II) production was observed and initial cellular FeRR only slightly decreased as a function of pressure. This suggests that the respiratory chain was not immediately affected by pressure. This agrees well with the stability of terminal oxidases of respiratory chain that are active up to 200 and 125 MPa, respectively, in the piezophilic bacteria *S. violacea* DSS12 and *Photobacterium profundum* SS9 (Chikuma et al., 2007; Tamegai et al., 2012). Proteins are stable at least up to pressures of 100–200 MPa (Silva and Weber, 1993). While ATP production is not significantly inhibited in *Streptococcus faecalis* incubated at 41 MPa, the ATP demand increases with increasing pressure (Matsumura and Marquis, 1977). Catabolic processes are generally less sensitive to pressure than anabolic processes (Pope and Berger, 1973). We therefore hypothesize that ATP demand in *Sp* LT13a might also increase with increasing pressure. That demand could therefore be fulfilled up to 40 MPa as long as growth occurs and a steady-state population is maintained. Above that pressure, only cells in the higher cell density experiments were active. As high cell density might induce a “resting state” for cells which requires less maintenance energy than more active cells, even if levels increase with increasing pressure. Cells would thus be coping with pressure for a longer time, and thus have a higher pressure limit for activity.

### ENVIRONMENTAL SIGNIFICANCE OF *Shewanella profunda* LT13a

The genus *Shewanella* is widespread in sedimentary environments and is characterized by a wide metabolic diversity (Kato and Nogi, 2001; Lauro and Bartlett, 2007; Fredrickson et al., 2008). We show here that *Sp* LT13a is potentially active in the range 4–37°C and 0.1–120 MPa. In terms of deep environments, this translates into conditions found in the ocean down to the deepest trenches (e.g., Mariana Trench, 11000 m water depth, 110 MPa), or in sediments, rocks and aquifers at depths of up to >1 km following a normal geothermal gradient. The apparent activation energy of 50.3 ± 0.8 kJ mol^-1^ at ambient pressure was close to values estimated for Fe(III) reduction in freshwater environments, i.e., 43.6 kJ mol^-1^ in rhizosphere sediments and 42.3–45.5 kJ mol^-1^ in pit lake sediments (Roden and Wetzel, 1996; Meier et al., 2005). The upper pressure limits for Fe(III)-reducing activity of *S. profunda* LT13a and of *S. oneidensis* MR-1 were similar, 120 and 110 MPa respectively (Picard et al., 2012), even though the first is piezophilic and the second is piezo-sensitive. Only little information about the physiology of *Sp* LT13a is available (Toffin et al., 2004) and its genome has not been sequenced. However information on *S. piezotolerans* WP3 could provide some clues about the adaptations of Fe(III)-reducing deep-sea *Shewanella* strains to deep-sea environments (Wang et al., 2008, 2009; Wu et al., 2013). Strain WP3 reduces Fe(III) at HP (Wu et al., 2013) and average FeRR per CFU follow similar trends as a function of pressure in *Sp* LT13a and in *S. piezotolerans* WP3 (**Figure 5**), suggesting that deep-sea strains might have a similar response to pressure. The mtrABC/omcA gene cluster, responsible for metal reduction, is present in WP3 (Wang et al., 2008). Strain WP3 has 55 putative c-type cytochrome genes, suggesting a great metabolic diversity, similarly to MR-1 (Wang et al., 2008). Some of these cytochromes could potentially be adapted to pressure. It appears that MR-1 evolved after WP3 (Wang et al., 2008), explaining why MR-1 has also a wide range of pressure for metabolic activity. Finally strain WP3 increases its levels of low-melting point fatty acids [branch-chained fatty acids (BCFA), monounsaturated fatty acids (MUFA), and eicopentaenoic acid (EPA)] with increasing pressure (Wang et al., 2009). MUFA and BCFA dominate the fatty acid composition of *Sp* LT13a, however EPA is not produced, at least at atmospheric pressure (Toffin et al., 2004). Deep-sea strains seem thus well adapted to subsurface environments.

Although cell concentrations used in this study are unrealistic for deep subsurface environments, this study reveals some aspects of cell metabolism in conditions where cells might be in a maintenance or “resting” state. In the deep biosphere, microbial cell populations are maintained over geological times, although the biomass turnover is very slow (Colwell and D’Hondt, 2013). Many questions concerning microbial physiological states and energy metabolism in subsurface environments remain unanswered (Hoehler and Jorgensen, 2013). For example, it is still unknown how to distinguish between active and resting cells, or what define maintenance processes in microbial cells. Laboratory studies of subsurface isolates might help to understand better energy regulation and use under *in situ* pressure and temperature conditions.

## Conflict of Interest Statement

The authors declare that the research was conducted in the absence of any commercial or financial relationships that could be construed as a potential conflict of interest.
